# ^[18F]^FDG Positron emission tomography with whole body magnetic resonance imaging (^[18F]^FDG-PET/MRI) as a diagnosis tool in Schwannomatosis

**DOI:** 10.1186/s13023-021-01680-0

**Published:** 2021-01-28

**Authors:** I. Gallais Sérézal, S. Ferkal, L. Lerman, S. Mulé, B. Funalot, P. Wolkenstein

**Affiliations:** 1 Service de Dermatologie, Centre Hospitalo-Universitaire de Besançon, Besançon, France; 2 Département de Génétique, Hôpital Henri-Mondor, Assistance Publique-Hôpital Paris (AP-HP), Créteil, France; 3 INSERM, Centre D’Investigation Clinique 006, centre de référence des neurofibromatoses, Hôpital Henri-Mondor, Assistance Publique-Hôpital Paris (AP-HP), Créteil, France; 4 Service de Médecine Nucléaire, Hôpital Henri-Mondor, Assistance Publique-Hôpital Paris (AP-HP), Créteil, France; 5 Service de Radiologie, Hôpital Henri-Mondor, Assistance Publique-Hôpital Paris (AP-HP), Créteil, France; 6 Service de Dermatologie, Hôpital Henri-Mondor, Assistance Publique-Hôpital Paris (AP-HP), Créteil, France

**Keywords:** Schwannomatosis, Diagnosis, ^[18F]^FDG-PET, MRI

## Abstract

Schwannomatosis is a rare autosomal dominant genetic syndrome characterized by the presence of multiple schwannomas. The main symptom is neurogenic pain. The diagnosis requires the presence of several schwannomas and whole-body ^[18F]^FDG-PET/MRI might help detect extra schwannomas in patients when the diagnosis is uncertain. Among the 25 patients treated for Schwannomatosis in our tertiary center, three men and two women had had a ^[18F]^FDG-PET/MRI performed, and the number of schwannomas detected by ^[18F]^FDG-PET/MRI outnumbered the number of schwannomas suspected during the clinical examination. The majority of schwannomas exhibited a radiolabeling (median of 66.7%, range 28–93%). Our findings show that ^[18F]^FDG-PET/MRI could prove useful when suspecting schwannomatosis to accelerate diagnosis and offer optimal care to patients.

Schwannomas are benign tumors of the nerve sheaths. Most of them are solitary lesions, but multiple schwannomas develop in genetic disorders like neurofibromatosis type 2 (NF2) and schwannomatosis. Schwannomatosis is an autosomal dominant genetic syndrome characterized by the presence of multiple schwannomas, and less often meningiomas. The main symptom of schwannomatosis is neurogenic pain but other neurological manifestations are possible, such as muscle atrophy and weakness [1, 2]. Transformation into malignant tumors is extremely rare [3].

An optimal evaluation of the number of lesions is capital in the diagnosis of schwannomatosis since it requires the identification of two or more schwannomas [1]. Also, patients must have no evidence of bilateral vestibular schwannomas on magnetic resonance imaging scan (MRI), no first-degree relative with diagnosed NF2, and no known constitutional *NF2* mutation. Whole-body imaging has developed in recent years, and both MRI and ^[18F]^FDG Positron emission tomography (PET) -computed tomography (CT) can help detect asymptomatic lesions. Considering that MRI allows better detection of neural tumors than CT [4] and that schwannoma often exerts hypermetabolism on PET-CTs [5, 6], the combination of both ^[18F]^FDG-PET and MRI methods could prove useful in schwannomatosis. Hence, the interest of ^[18F]^FDG-PET/MRI was tested and then reported in several observations of schwannomatosis [7–9]. In 2015, a study evaluating 153 schwannomas in 13 patients performed ^[18F]^FDG-PET/MRI and showed that the average maximum standardized uptake value (SUV_max_) of the schwannomas was 6 [10]. This study confirmed that ^[18F]^FDG-PET/MRI did not apply to the discrimination between benign and malignant tumors in schwannomatosis as ^[18F]^FDG-PET hypermetabolism is uncorrelated to a malignant process but suggested that it might help detect extra schwannomas in cases of uncertain diagnosis.

To test this hypothesis, we retrospectively collected data on ^[18F]^FDG-PET/MRI performed at a single tertiary center in patients with schwannomatosis.^[18F]^FDG-PET/MRI are routinely performed by injecting 4.5 MBq/kg of ^[18F]^FDG one hour before the simultaneous acquisition of the PET and the MRI sequences of the whole body. The acquisition includes the simultaneous acquisition of PET using motion correction and two MR sequences: 3D-T1-Dixon attenuation correction sequences (MRAC) and diffusion-weighted imaging. PET images were reconstructed with and without MR attenuation correction. Secondly, coronal T2-weighted fat-suppressed images were acquired. Both a radiologist and a nuclear radiologist read the images. Statistical analysis was performed with Graphpad prism, using Wilcoxon test to compare the number of schwannoma detected by the different methods. The asterisk * corresponds to a p-value under 0.05.

Of the 25 patients with a confirmed diagnosis of schwannomatosis, three men and two women had had a whole-body ^[18F]^FDG-PET/MRI (Table 1). The median age was 23 years (range 18–35) and the median delay between the start of symptoms and the diagnosis was 2 years (range 0–14). Two patients had a segmental form. All patients fulfilled the diagnostic criteria of schwannomatosis, had no family history of neurofibromatosis, and developed at least two histologically-confirmed schwannomas. The median SUV_max_ was 2.5 (range 1.7–6.4). In all five patients, the number of schwannomas detected by ^[18F]^FDG-PET/MRI outnumbered the number of schwannomas suspected during the clinical examination (Fig. 1). We could confirm that the majority of schwannomas exhibited a radiolabeling (median percentage of lesions detected for each patient 66.7%, range 28–93%) (Fig. 2). The two imaging modalities appear to be complementary in the work-up of schwannomatosis, as each technique offers the possibility of identifying schwannomas that were not detected by the other imaging modality (Figs. 3, 4).

**Table 1:** Patient characteristics

	Patient				
	1	2	3	4	5
*Presentation*					
Age at diagnosis	35	18	23	19	23
Sex	M	M	F	M	F
Diagnostic delay (year)	2	0	0	7	14
Pain (1/0)	1	1	1	1	1
Number of symptomatic body sites (N)	1	1	4	2	1
Segmental form (1/0)	0	1	0	0	1
*Diagnosis*					
Two or more non-intradermal tumors suggestive of schwannomas (1/0)	1	±	1	1	1
History of bilateral vestibular schwannomas (1/0)	0	0	0	0	0
Family history of schwannomatosis (1/0)	0	0	0	0	0
Histology-proven schwannomas (N)	2	> 2	> 4	2	> 3
*Genetic*					
Mutation	No mutation found in *SMARCB1/INI1*, *LZTR1*, *NF2*	N/A	p.Tyr35del gene *SMARCB1/INI1*	N/A	p.Arg362* gene *LZTR1*

N/A, not available; M, male; F, female. 1 = present, 0 = absent. ± refers to a patient in which it was unclear from the medical records whether the patients’s schwannomas were subcutaneous or not. Le symbol “*” represents a stop codon. Del, deletion; p, protein

**Fig. 1:**
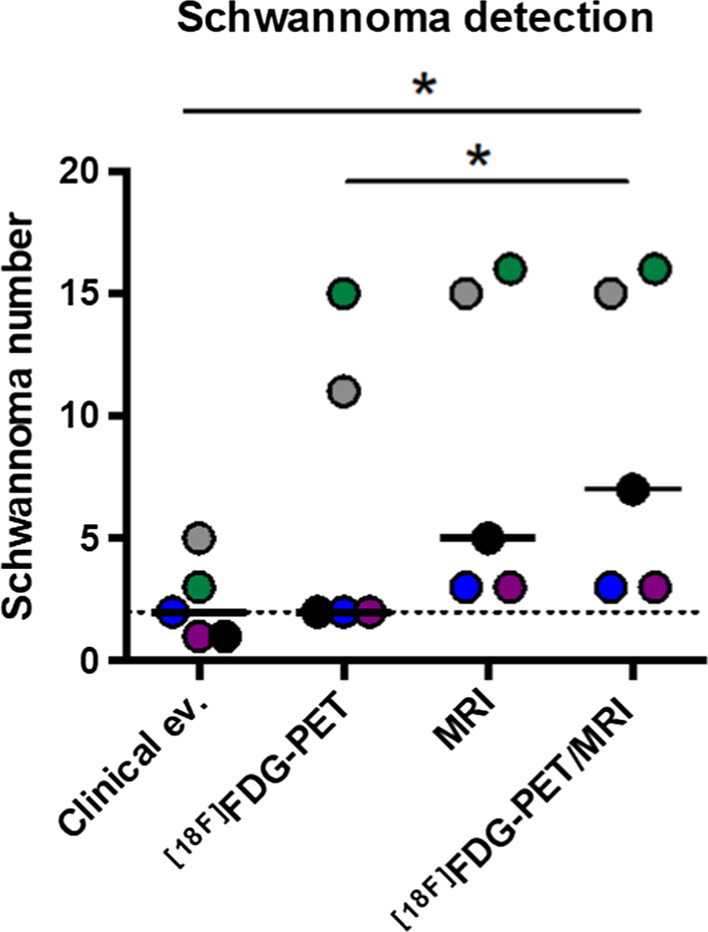
Comparison of the number of schwannomas detected by the different methods. Each color represents a single patient. The dotted line represents the median detection by clinical examination alone. Bars show medians for each group. The * is for p < 0.05, paired T test. Clinical ev., clinical evaluation

**Fig. 2:**
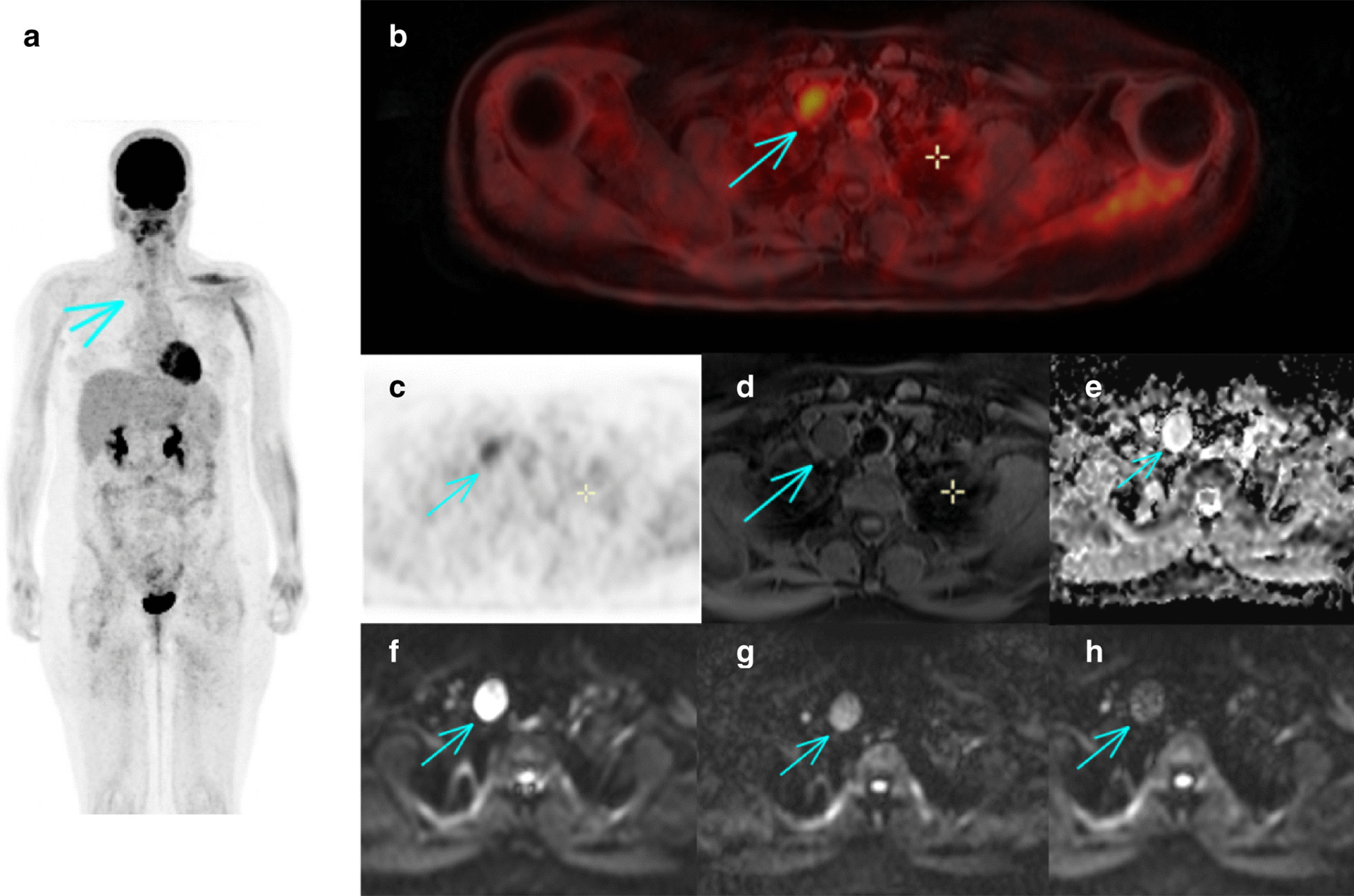
Example of a schwannoma detected by both MRI and ^[18F]^FDG-PET. From top left to down right: **a** Coronal maximum intensity projection (MIP) ^[18F]^FDG-PET image, **b** axial fused TEP/T1w image, **c** axial ^[18F]^FDG-PET image, **d** axial T1-weighted (T1w) fat-suppressed image, **e** axial apparent diffusion coefficient (ADC) image and **f**–**h** axial diffusion-weighted images with 3 b values (50, 400 and 800 s/mm^2^). The arrows indicate the same schwannoma in the different images

**Fig. 3:**
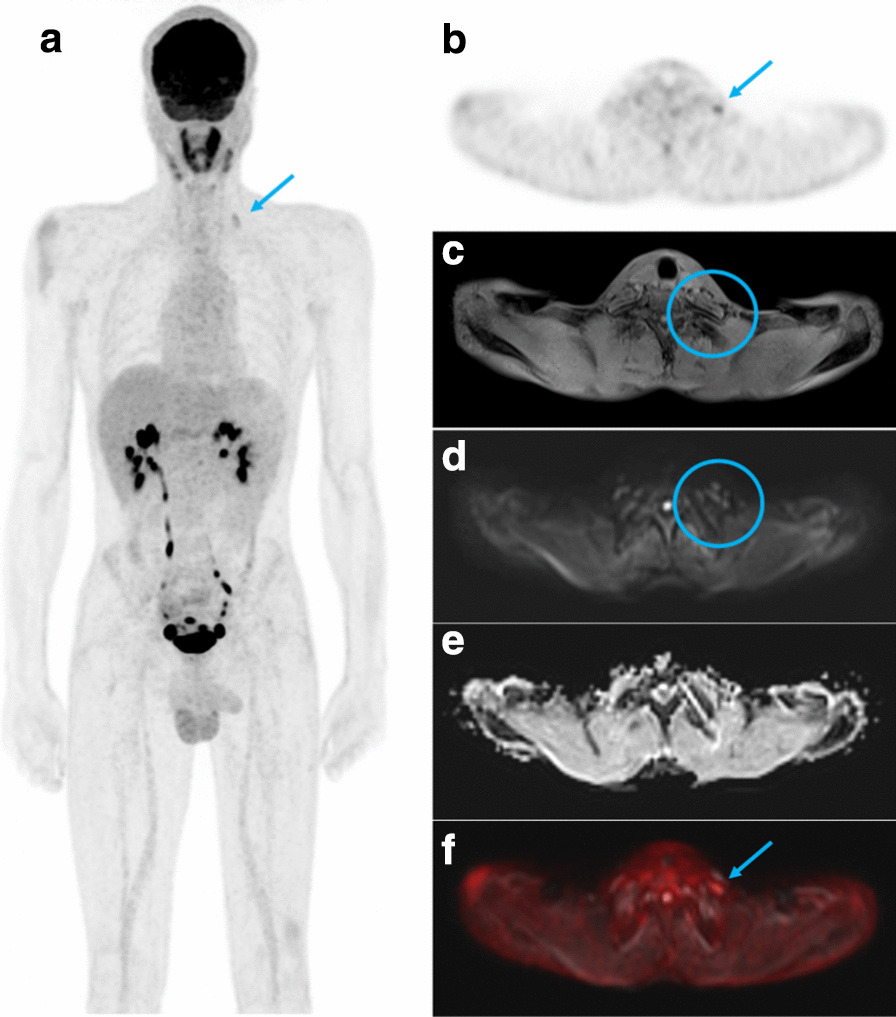
Example of a schwannoma detected by ^[18F]^FDG-PET but not MRI. Left-sided supraclavicular schwannoma (arrows) visible on **a** coronal maximum intensity projection (MIP) ^[18F]^FDG-PET image and **b** axial ^[18F]^FDG-PET image. Conversely, the lesion was not detected on **c** axial T1-weighted fat-suppressed image, **d** axial b = 800 s/mm^2^ diffusion-weighted (DW) image and **e** axial apparent diffusion coefficient (ADC) image (circles). **f** Axial fused ^[18F]^FDG-TEP and b = 800 s/mm2 DW image

**Fig. 4:**
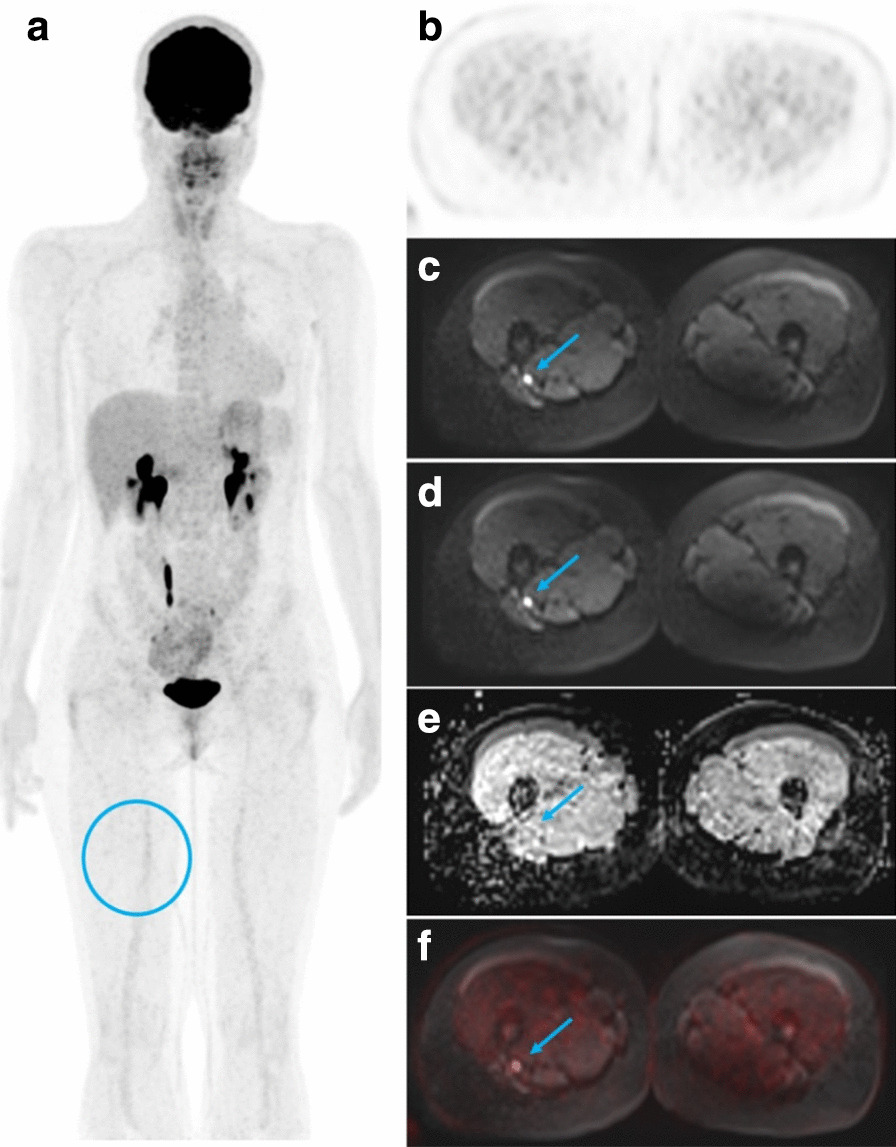
Example of a schwannoma detected by MRI but not by ^[18F]^FDG-PET. Right-sided sciatic nerve schwannoma (arrows) not visible on **a** coronal maximum intensity projection (MIP) ^[18F]^FDG-PET image and **b** axial ^[18F]^FDG-PET image (circles). Conversely, the lesion was detected on MRI as a high signal intensity focal area on **c**, **d** axial diffusion-weighted (DW) images with low and high values (50 and 800 s/mm^2^), an intermediate signal intensity on **e** axial apparent diffusion coefficient (ADC) image. **f** Axial fused ^[18F]^FDG-TEP and *b* = 800 s/mm^2^ DW image

In conclusion, our findings show that ^[18F]^FDG-PET/MRI could prove useful in accelerating the diagnosis of schwannomatosis and in offering an optimal follow-up to patients. To better investigate the benefit of ^[18F]^FDG-PET/MRI as a diagnostic tool, a prospective evaluation of patients suspected to have schwannomatosis before they fulfill the diagnosis criteria would be of great interest.

## Data Availability

Not applicable.
